# Ankyloglossia in Children, a Cause of Obstructive Sleep Apnoea: Case Report of Paediatric Ankyloglossia and Sleep Apnoea: DISE Resolves the Mystery

**DOI:** 10.3390/children11020218

**Published:** 2024-02-08

**Authors:** Johanna Ximena Valderrama-Penagos, Laura Rodríguez Alcalá, Guillermo Plaza, Peter Baptista, Maria Teresa Garcia Iriarte, Eduardo J. Correa, Carlos O’Connor-Reina

**Affiliations:** 1 Department of Otorhinolaryngology, Hospital Quiron Salud Marbella, Av. Severo Ochoa 22, 29603 Marbella, Spain; 2 Department of Otorhinolaryngology, Hospital Fuenlabrada, Universidad Rey Juan Carlos I, 28032 Madrid, Spain; 3 Department of Otorhinolaryngology, Clinica Universitaria de Navarra, 31008 Pamplona, Spain; 4 Department of Otorhinolaryngology, Hospital Universitario Virgen de Valme, 41014 Seville, Spain; 5 Department of Otorhinolaryngology, Hospital La Linea, 11300 La Linea de la Concepción, Spain

**Keywords:** ankyloglossia, drug-induced sleep endoscopy, frenulectomy, sleep apnoea

## Abstract

Tongue mobility is an obstructive sleep apnoea (OSA) marker and myofunctional therapy (MFT) target. For this reason, all paediatric patients with sleep-disordered breathing should require a combined functional assessment from an ear, nose, and throat (ENT) specialist and a phonoaudiologist to confirm or rule out the presence of ankyloglossia. To our knowledge, this is the first case of a 13-year-old girl diagnosed with severe OSA and a significant decrease of 94% in her apnoea index (AI), requiring frenotomy with an immediate postoperative change in the tongue position. A drug-induced sleep endoscopy (DISE) was performed before and immediately postfrenotomy, and the anatomical changes provoked by this surgery during sleep were confirmed for the first time.

## 1. Introduction

Most obstructive sleep apnoea (OSA) patients share a common physiopathology: the narrowing of the upper airway, mainly at the retropalatal and retroglossal levels. This can be due to increased soft tissue from fatty deposits and tissue hypertrophy owing to the chronic inflammatory process, a volumetric reduction in the facial skeletal framework caused by craniofacial characteristics related to genetic or environmental factors, alterations in neuromuscular tone, or a combination of these factors. OSA has a multifactorial aetiology, characterized by the abnormal (total or partial) collapse of the upper airway during sleep, producing disturbances in ventilation and sleep architecture [1]. It occurs in 3–10% of children with two prevalence peaks, one in the 2–8-years-of-age range and another in adolescence, related to weight gain.

The outcomes of multiple investigations in the paediatric population reveal a pattern: early dysfunction involving abnormal nasal breathing, sucking, and chewing, leading to progressive dysmorphia, which results in increased upper airway collapsibility during sleep [2].

Several risk factors for developing paediatric OSA have been described, including obesity, adenoid and tonsillar hypertrophy, allergic rhinitis, and septal deviation. Short lingual frenulum is also a risk factor [1]. According to the guidelines of the American Academy of Otolaryngology—Hea and Neck Surgery (AAO-HNS), ankyloglossia is defined as a condition of limited tongue mobility caused by a restrictive lingual frenulum that has an incidence of 2.8–10.7% [3]. The condition described is related to X-linked cleft palate (CPX), which results from mutations in the TBX22 gene [2]. It is also associated with patients with Beckwith–Wiedemann syndrome, orofacial digital syndrome, cleft palate, and Optiz syndrome [4].

Ankyloglossia does not allow the tongue to rest on the palate, meaning that during sleep, the tongue falls towards the pharynx and obstructs the airway, which explains the appearance of OSA [1]. The balance between the tongue and the buccinator muscles is responsible for the normal development of the maxillary and mandibular arch. The stimuli of abnormal bone growth, the absence of nasal breathing, and hypotonia with the consequent development of oral breathing associated with ankyloglossia are responsible for abnormal orofacial development, thus increasing the risk of collapse in the upper airway.

An ear, nose, and throat (ENT) specialist should consider a functional assessment for patients with OSA, including examining the lingual frenulum [4]. However, our group demonstrated [5] that the evaluation of a short lingual frenulum was not included in any otolaryngology clinical guidelines. Therefore, the diagnosis of sleep breathing disorder (SBD) in patients with ankyloglossia is delayed, unlike in children with tonsillar hypertrophy [4]. The following of all these patients must be evaluated: the craniofacial region, breathing, elongated face shape, the coexistence of retrognathia, dental crowding, high and narrow palate, tongue posture, and the length and mobility of the lingual frenulum [5].

Paediatric drug-induced sleep endoscopy (DISE) [6] is considered in children with persistent, postadenotonsillectomy obstructive sleep apnoea who refuse or fail positive pressure therapy. However, many questions regarding the timing of DISE, patient selection, ideal scoring method, and threshold for operative intervention remain unanswered as no controlled studies of DISE-assisted surgery in children have been conducted. Up to now, there is no case reported in a paediatric OSA patient showing anatomic changes after a therapeutical intervention. DISE is commonly used to explain anatomical findings in OSA patients without a reasonable explanation of their disease [7] and, in our experience, also to improve adherence to myofunctional therapy [8,9]. We introduce the first OSA paediatric case, where Ankyloglossia surgery combined with MT resolves the disease, demonstrated by DISE performed pre- and postoperatively.

## 2. Case Description

A 13-year-old female patient with sleep problems witnessed apnoeas and slight snoring. She had a level III sleep study (nocturnal respiratory polygraphy was performed using an Embletta portable diagnostic system (ResMed, Sydney, Australia) according to the technical specifications of the American Academy of Sleep Medicine) [10]. Measurements were obtained using a snoring sensor, nasal thermistor, and nasal pressure cannula to register airflow; thoracic and abdominal belts to assess ribcage and abdominal movements; electrocardiography; actigraphy to detect body position; oxygen saturation; and heart rate (in February 2022) with the result of apnoea–hypopnoea index (AHI) 17.3/hour, 14 obstructive apnoeas, 15 central apnoeas, 10 mixed apnoeas, and 99 hypopnoeas. Oxygen desaturation index (ODI) was 2.4; T90 was 0.1%; mean oximetry was 95%; and nadir was 83%. She scored 18 on the Epworth sleepiness questionnaire (ESS). On physical evaluation, she presented BMI 21 Kg/m^2^, moderate hypertrophy of the turbinates, nonadenotonsillar hypertrophy, normal palate, Angle 1 classification, teeth malposition, and the unique significant finding was the ankyloglossia. This was evaluated following the Marchesani protocol [11]. The measurement was taken during the maximum opening of the lower left incisor to the upper left incisor. Then a measurement was taken with the mouth wide open with the tip of the tongue touching the incisor papilla. This measure was considered normal if the difference between the two was <50%. Our protocol functional assessment [8] included measurements with the Iowa Oral Performance Instrument (IOPI), and our results were maximum tongue strength 36 kilopascal (Kps) (normal 65 Kps) and maximum buccinator strength 24 Kps (normal 35 Kps).

**Figure 1:**
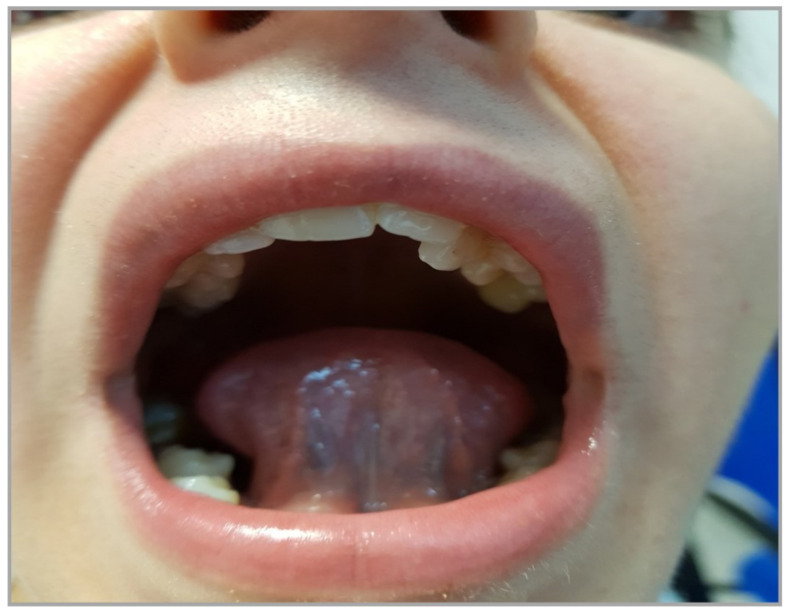
Ankyloglossia associated with teeth malposition.

Many otolaryngologists have examined her without any objective finding to explain the disease. The patient was referred to speech therapy, and in the assessment, a frenotomy was considered to manage the short lingual frenulum (Figure 1).

**Figure 2:**
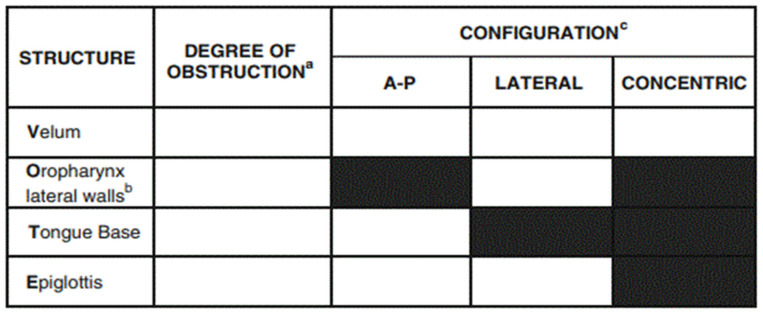
The surgical protocol used to code the DISE results according to the VOTE scale is as described in reference. ^a^ Degree of obstruction has one number for each structure: 0, Noobstruction (no vibration); 1, Partial obstruction (vibration); 2, Complete obstruction (collapse); X, Not visualized. ^b^ Oropharynx obstruction can be distinguished as related solely to thetonsils or including the lateral walls, with or without a tonsillarcomponent. ^c^ Configuration noted for structures with degree of obstructiongreater than.

Before surgery, drug-induced sleep endoscopy (DISE) was performed using an inhaled anaesthetic agent, allowing for intravenous access to be obtained. The inhalation agent is then discontinued, and a propofol infusion dosed appropriately for the child’s age and weight was used for the remainder of the procedure. We visualized anteroposterior collapse due to tongue base with secondary lateral epiglottic collapse and twisted epiglottis. Vote classification [12] was used to evaluate it, resulting in V1APO1LT2APE2LS. Vote classification is the most extended classification used to assess the results from a DISE. The configuration of obstruction can be described as anteroposterior (typically anterior structures moving posteriorly against the posterior pharyngeal wall), lateral (laterally located structures moving towards the centre of the airway), or concentric (combination of the former two) [12] (Figure 2).

Then, the patient underwent turbinoplasty combined with lingual frenotomy under sedation, and a new DISE was performed immediately after the operation, showing the adequate position of the tongue with an improvement in retroglossal collapse and complete decrease in epiglottic collapse, with an adequate response to chin lift and Esmarch manoeuvres, and vote classification improved to V1APO1T0PE0 (see Video S1). The postoperative period was uneventful, and the patient and, since the first postoperative day, her family reported improved sleep patterns and decreased daytime sleepiness (see Video S2).

The patient started to perform myofunctional therapy, and a new sleep study was requested two months after the procedure. Postoperative control in August 2023 significantly improved her OSA with an AHI of 4.2/hour (1 obstructive apnoea—3.2 hypopnoeas), 0 central apnoeas, 0 mixed apnoeas, and 30 hypopnoeas. ODI and T90 were 0%, mean oximetry was 98%, and nadir was 91%. ESS score was 24, her IOPI tongue measurement was 52 Kps, and her buccinator evaluation was 30 Kps.

Presently (24 December 2023), no recurrence of symptoms has occurred, with similar results obtained in a new sleep study (see Table 1). She is now under orthodontic treatment by a specialist.

## 3. Discussion

This is the first paediatric DISE where anatomical changes after a frenotomy are objectively documented by performing another immediate postoperative DISE.

The tongue is a dynamic organ that affects breath, language, early weaning, breastfeeding, sucking, and swallowing, with a critical role in facial development and the oropharynx’s patency [4]. Lingual frenulum is a vestigial embryological element of mostly fibrous consistency, a product of the adhesion between the tongue and floor of the mouth that keeps the hard and soft tissue balanced to promote adequate growth. During tongue formation, cells of the lingual frenulum undergo gene-mediated apoptosis and migrate distally to the medial dorsum of the tongue; incomplete or absent migration results in the development of ankyloglossia [3,4].

Ankyloglossia, commonly described as “ankyloglossia”, is a short or altered insertion of the lingual frenulum that restricts the tongue’s mobility. It also hurts orofacial development (dentofacial anomalies, abnormal growth of the maxilla with a narrow palatine arch, and imbalanced mandible growth). It modifies the respiratory function because of the reduced calibre of the upper airway with the risk of collapsibility during sleep [3,5]. Ankyloglossia generates the use of additional facial muscles and modifies the cervical muscles and is associated with incorrect head posture, nasal obstruction, and oral breathing [3].

The untreated short lingual frenulum is associated with OSA. In ankyloglossia, the tongue rests on the mouth’s floor, contrary to its normal position, resting against the upper incisors and hard palate [4]. Oral breathing, one of the nocturnal symptoms of children with SBD and particularly with OSA, was more prevalent in patients with reduced mobility of the tongue, and compared with healthy children, they were also more prone to developing hypotonia, as measured by IOPI [5]. This confirms that the position of the tongue has special importance in the development of OSA.

Huang et al. concluded that the assessment of frenulum length should be performed in all children with OSA, as well as OSA screening and surgical management in all children with ankyloglossia as soon as possible [13]. Guilleminault et al. [1] reported a relationship between OSA and ankyloglossia in 150 children with SBD and found short lingual frenulum in 64 patients associated with oral breathing. Brozek et al. [14] reported that children with positive questionnaires for high risk of OSA had a short ankyloglossia and, more likely, a high-arched palate, which is also a risk factor for OSA Additionally, Man Yuen [15] collected data from 82 paediatric patients and found a high prevalence of OSA in patients with short lingual frenulum. These findings have also been described by Guilleminault, who reported a strong association between high-arched palate and children with ankyloglossia compared with the control, with statistically significant results of 80% vs. 8.75%, respectively [1].

The removal of ankyloglossia in a patient with adequate muscular strength in the tongue allows for the oral cavity to be spread and collapse to be avoided. Under normal conditions, the tongue contacts the hard palate and does not invade the retroglossal space [1,4]. The tongue’s mobility is affected by the length of the lingual frenulum and the elasticity of the floor of the mouth, and patients with reduced length of the frenulum could have normal mobility of the tongue and enough force to ensure the normal development of the maxilla [9]. In patients with a lack of strength in the tongue muscles, an upward vector cannot be established with subsequent difficulty for stimulation of the intermaxillary synchondrosis, affecting bone growth and favouring the development of a V-shaped maxilla, creating a narrower nasal floor, affecting the oropharyngeal space, and increasing the risk of nasal obstruction, which once again explains the relationship between lingual frenulum and OSA.

Frenotomy in children is a safe and well-tolerated procedure, although there is no consensus on the ideal or suitable specific surgical technique. The surgical approach to ankyloglossia is referred to intermittently in the literature. Still, the term “frenotomy” or “frenulotomy” is routinely used for procedures performed in children in which a simple incision of the ankyloglossia is made. Frenotomy is an incision of the lingual frenulum with a reorganization of the tissue, and frenectomy is used to remove the lingual frenulum. In 2023, our group suggested that lingual frenulum surgery (frenotomy) was an option for patients with OSA that can improve the results of MFT because ankyloglossia limits the success and adherence to this therapy [5].

MFT improves muscle tone and position of the tongue and decreases respiratory events. The current literature demonstrates that myofunctional therapy decreases the apnoea-hypopnoea index by approximately 50% in adults and 62% in children [16,17]. For this reason, our protocol recommends that all patients who will be managed with frenotomy, including children, should be evaluated by speech therapists and undergo MFT before and after the surgical procedure to achieve better results [5]. On the other hand, IOPI measurements allow patients to objectively evaluate their tongue strength and enable the therapist to select patients who should undergo MFT [18] appropriately. Villa et al. [19] pioneered this instrument, establishing a relationship between tongue strength and SDB in paediatric patients. They performed a RCT between a group of 36 children treated with MT versus a group 18 children with sham therapy. After performing oropharyngeal exercises, the MT group reduced oral breathing (83.3 vs. 16.6%, *p* < 0.0002) and lip hypotonia (78 vs. 33.3%, *p* < 0.003), restored normal tongue resting position (5.6 vs. 33.4%, *p* < 0.04), and significantly increased mean tongue strength (31.9 ± 10.8 vs. 38.8 ± 8.3, *p* = 0.000). Moreover, mean oxygen saturation increased (96.4 ± 0.6 vs. 97.4 ± 0.7, *p* = 0.000), and the oxygen desaturation index decreased (5.9 ± 2.3 vs. 3.6 ± 1.8, *p* = 0.001).

The frenotomy technique used consists of a three-step procedure. First, we perform DISE, followed by frenotomy under sedation using local anaesthesia to carry out a cold dissection and bipolar coagulation. Finally, a new DISE is essential to assess postoperative changes in the upper airway [9]. Complications of lingual frenotomy include haemorrhage, airway obstruction, injury to salivary structures, and abnormal scarring [9]. We have not found any significant complications to date, so we recommend this procedure due to its safety and feasibility [20].

Despite the results of multiple studies, the AAO guidelines suggest insufficient evidence to associate ankyloglossia with OSA [4]. In fact, it is considered that, in some cases, ankyloglossia can prevent subsequent collapse, and frenotomy could worsen OSA [9]. This point is made based on two case reports with insufficient evidence. On the other hand, this group has described that the release of the tongue with correct muscle tone can prevent collapse and does not lead to glossoptosis. Furthermore, the tongue can rest in its normal position on the palate, as evidenced in this case.

In the lecture by Professor Dr. Eric Kezirian in 2020, available on his YouTube^®^ Channel [21], he insinuated that ankyloglossia surgery should not be considered as treatment for OSA. We demonstrated that it can be used as a potential tool to treat OSA in select cases like this. We know the risk of universalizing this treatment due to its feasibility and low morbidity among other therapists not focused on OSA. It is essential to notice that an ENT specialist should use frenotomy after excluding other anatomical potential causes of OSA.

Nose procedures have been shown to be ineffective for reducing the apnoea–hypopnoea index [22,23], so we are aware that the improvement obtained in this patient derives mainly from frenotomy.

We are aware that some patients with true ankyloglossia maintain normal tongue posture, while others with myofunctional problems may have their tongues resting on the floor of the mouth without ankyloglossia. Several factors contribute to this, including muscle tension, functional habits, oral cavity space, orthodontic and maxillofacial issues, and upper airway obstruction. Tongue resting position is a multifactorial problem and should be evaluated under a multispeciality team.

We believe this technique of DISE–frenotomy–DISE is a useful procedure to assure the benefits of surgery, allowing for an evaluation of further complementary treatments if required. Furthermore, this technique increases adherence to MFT, providing the patient with visual feedback about the anatomical reasons for their disease [8]. We are considering the predictive factors involved in this procedure’s success as we know that not all patients with Ankyloglossia are candidates for this procedure. We believe that the tongue must have space to reach to its optimal position in the oral cavity, and postoperative rehabilitation with a speech therapist will always be recommended [9].

## 4. Conclusions

Functional ankyloglossia examination should always be performed in all paediatric SBD patients, and it must be suspected when there are no other anatomical possible causes of OSA. DISE performed pre- and postfrenotomy is useful for evaluating anatomical changes and future therapeutic measures. We wish to emphasize with this case report the importance of collaboration in a multidisciplinary sleep unit. Procedures related to the ankyloglossia frenulum should only be performed after considering all other possible interventions by a combined approach.

## Figures and Tables

**Table 1:** Pre- and postoperative 3 and 6 months polygraphy results.

	Before Surgery	3 Months after Surgery	6 Months after Surgery
AHI	17.3	4.1	2.2
CA	15	0	0
AI	4.9	0.1	0
HI	12.4	4.1	2.2
SI	74.7	36.7	21.3
Min Sat O_2_	83%	91	93
ODI	2.4	0	0
Max PR	90	54	60
SpO_2_ (%)	95	98	99

AHI: apnoea–hypopnoea index, CA: central apnoeas, AI: apnoea index, HI: hypopnoea index, SI: snore index, Min Sat O_2_: minimal saturation O_2_, Max PR: maximal heart rate, SpO_2_ (%): average saturation O_2_.

## Data Availability

The data presented in this study are available in article and Supplementary Materials.

## Supplementary Materials

The following supporting information can be downloaded at: https://www.mdpi.com/article/10.3390/children11020218/s1, Video S1: DISE Pediatric frenotomy. Video S2: Mother’s history.
